# Potassium application to the cover crop prior to cotton planting as a fertilization strategy in sandy soils

**DOI:** 10.1038/s41598-020-77354-x

**Published:** 2020-11-23

**Authors:** Fábio Rafael Echer, Vinicius José Souza Peres, Ciro Antonio Rosolem

**Affiliations:** 1 Department of Agronomy, Universidade do Oeste Paulista, Raposo Tavares HWY, Km 572, Presidente Prudente, São Paulo 19067-175 Brazil; 2 São Paulo State University, Botucatu, São Paulo Brazil

**Keywords:** Plant sciences, Environmental chemistry, Environmental impact

## Abstract

*Urochloa* grasses are used as cover crops in tropical cropping systems under no-till to improve nutrient cycling. We hypothesized that potassium (K) applied to ruzigrass (*Urochloa ruziziensis*) grown before cotton in a sandy soil could be timely cycled and ensure nutrition, yield and quality of cotton cultivars with no need to split K application. Field experiments were performed with different K managements, applied to ruzigrass, to cotton grown after grass and without grass, or split as it is done conventionally. No yield differences were observed on K fertilized treatments. At 0 K, cotton yields were low, but they increased by 16% when ruzigrass was grown before, and short fiber content was lower when there was more K available. Ruzigrass grown before cotton increased micronaire as much as the application of 116 kg ha^−1^ of K without the grass. Fiber maturity was higher when K was applied to the grass or split in the grass and sidedressed in cotton. Growing ruzigrass before cotton allows for early K fertilization, i.e., application of all the fertilizer to de grass, since the nutrient is recycled, and cotton K nutrition is not harmed. Eventually K rates could be reduced as a result of higher efficiency of the systems.

## Introduction

Potassium (K) is the most abundant cation in plant tissue and is required by cotton in an amount similar to nitrogen (N). It plays a role in plant water relations through the regulation of stomata functioning, the rate of CO_2_ fixation, enzymatic activation, nutrient transport and homeostasis, stress tolerance, and eventually plant growth, fiber yield and quality^1^. In soils, K reaches the root mainly by diffusion^2^, which accounts for 72% to 96% of plant demand^3^, meaning that it is mostly bound to soil colloids. However, mass flow can contribute significantly when there is K available in the soil solution. In tropical sandy soils, which are often dominated by 1:1 clay minerals and Fe or Al oxides, low soil organic matter and low clay content result in low cation exchange (CEC) and low water retention capacity^4^. Hence, K binding to soil particles is limited in these soils. Under these conditions K in soil solution may be high for some time, increasing its movement by mass flow^5^, and also the risk of loss by leaching^6^. It has been shown that almost all K applied in excess to a tropical sandy soil is leached below the depth of 1.0 m^7^.

Using cover crops with vigorous, deep roots in cropping systems is an important tactic to avoid nutrient loss by leaching. This has been well documented for N^8^; however, there are not many studies on the effect of cover crops on K dynamics and cycling in cropping systems. Potassium is usually found in high concentrations in the vacuoles and cytoplasm of plant cells, and is not strongly bound to any structural molecules^9^. Hence, it will be easily washed out of the decaying cover crop residues with the first rains^10^ and will be available for the next crop^11^. Therefore, the use of cover crops, mainly grasses of the genus *Urochloa,* can be an effective tool in keeping the soil covered during fallow periods and increasing nutrient cycling in crop rotations as reported for soybean and maize^12^ in a clay soil. However, it is still unknown if similar results would be observed with cotton in sandy soils, since the possibility of K leaching is much higher. Potassium quick cycling by *Urochloa* could lead to higher flexibility in K fertilization of the system, allowing for an early application of the fertilizer, before crop planting, and optimizing farm operations^13^. The beneficial effect of early K fertilization on cotton yields in soils with medium and clay texture, and high CEC, is already known^14^; however, there is no information on sandy soils prone to K leaching, mainly considering cotton fiber quality.

In addition to the effects of cover crop residues on soil protection, crop rotations with grasses such as ruzigrass with deep, a vigorous root system, are important for developing a net of biopores in the soil profile, facilitating root growth of the next crop^15^. These grasses exude organic acids in the rhizosphere^16^, which can complex Al ions and increase the proportion of calcium (Ca), magnesium (Mg) and K in the soil CEC^17^, resulting in higher potassium use efficiency.

It has been shown that split K application, or the use of slow release K fertilizers is important in enhancing the efficiency of K fertilizers in sandy soils^8^. Therefore, applying at least part of the K fertilizer to a cover crop before cotton planting, can result in higher fertilizer use efficiency, because this nutrient would be cycled in the system and its release for the next crop would be slower than a single fertilizer application, avoiding loss in sandy soils and ensuring cotton nutrition, yield and quality. The objective of this work was to assess plant nutrition, fiber yield and quality of two cotton cultivars as affected by K fertilizer management and the use of ruzigrass as a cover crop.

## Results

Cotton cultivars showed similar performance and most of the characteristics showed no interaction with K management or season (Sup. 1), Therefore, the results were averaged over the two cultivars FM 913GLT and FM 983GLT.

### K on soil

**Figure 1:**
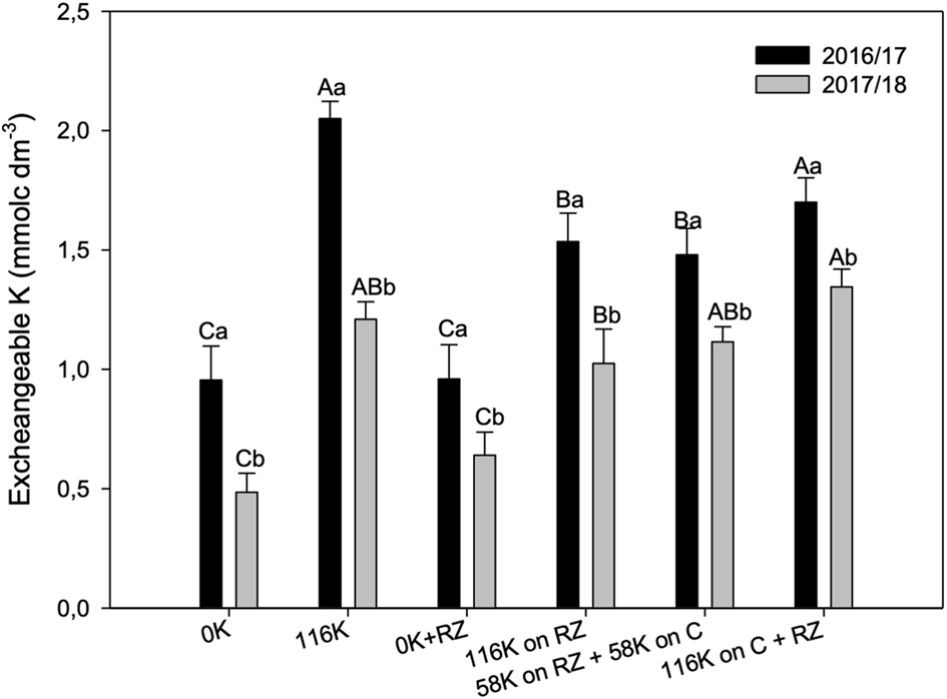
Exchangeable soil K in a 0–20 cm depth under potassium fertilization management after cotton harvest for two seasons. A > B for K management within each growing season and a > b for growing season within K management.

There was a significant interaction of the years with treatments, but the soil exchangeable K was on average higher in the first than in the second season (Fig. 1). By the end of the first year, exchangeable K was higher compared with the control plots where the fertilizer was applied, irrespective of ruzigrass. When K was applied to the grass, soil exchangeable K was increased compared with the control plots, but was lower than that observed in treatments without ruzigrass (Fig. 1). In the 2017/18 season, K fertilization increased soil exchangeable compared with the controls, except when the fertilizer was applied only to the forage (Fig. 1).

Excheangeable soil K was positivelly correlated with yield in both seasons, but the correlation coefficients, despite being significant (*P* < 0.05), were low, 0.42 and 0.53 in 2016/2017 and 2017/2018, respectively.

### K on leaf tissue

**Figure 2:**
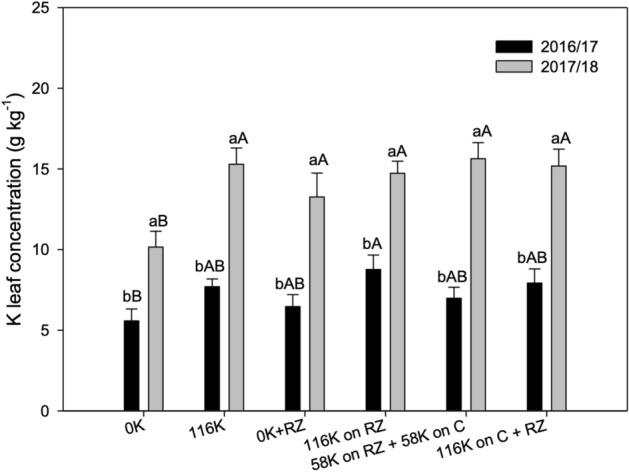
Potassium leaf content in cotton leaves under potassium fertilization management. A > B for K management within each growing season and a > b for growing season within K management.

Cotton grown after K fertilized RZ had higher K leaf concentration than unfertilized treatment without RZ in the 2016/17 season, and in 2017/18, this treatment had lower K on leaves than the others (Fig. 2). Leaf K was higher in the second season in all treatments and it can be explained by the better rainfall distribution in 2017/18.

### Yield and yield components

**Figure 3:**
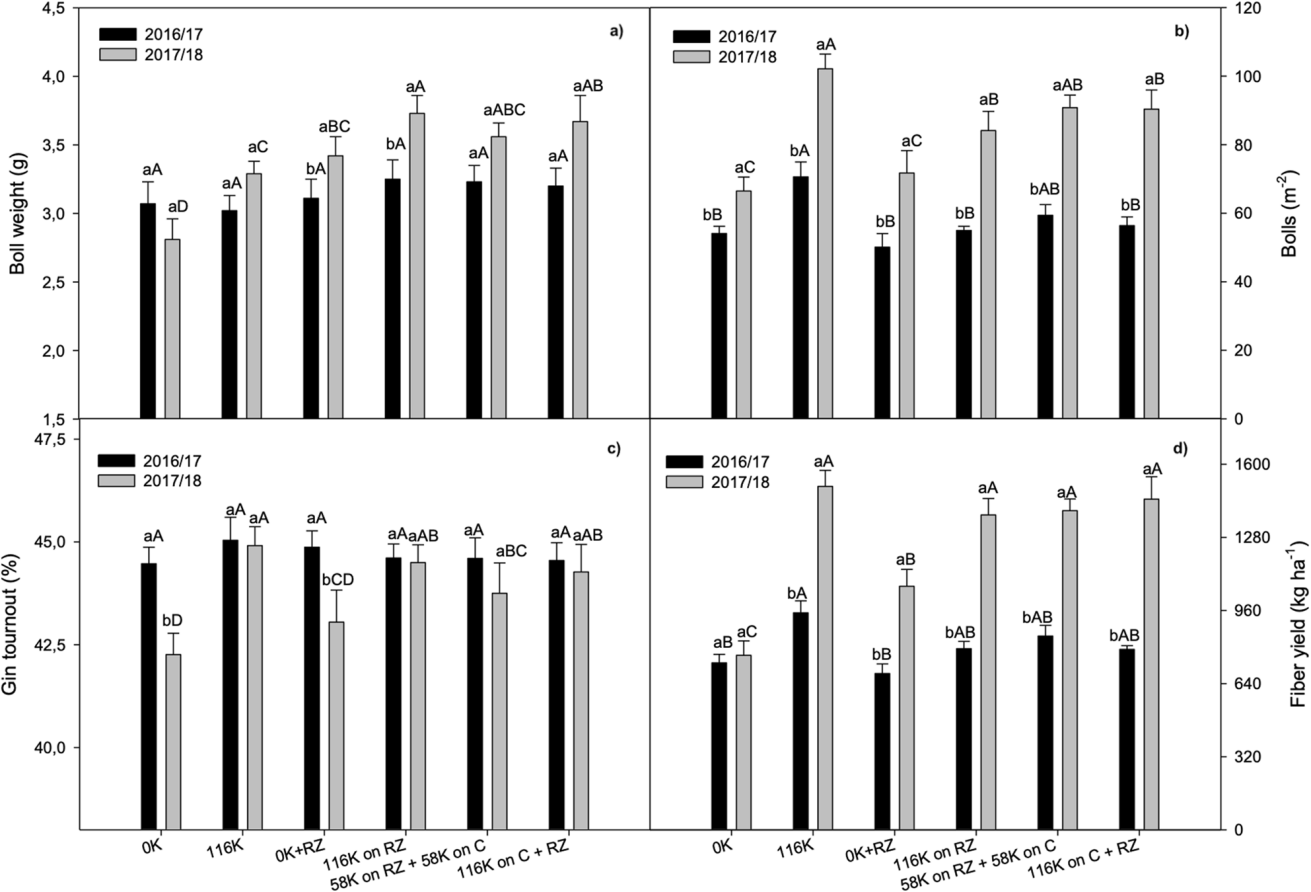
Cotton yield and yield components as affected by potassium fertilization management and season. A > B for K management within each growing season and a > b for growing season within K management.

In the first season, 2016/17, cotton boll weight was not affected by K management (Fig. 3a), but in the second season it was increased by K fertilization particularly in the presence of ruzigrass. Interestingly, even without K fertilizer, the average boll weight was higher after ruzigrass. The boll number differed each year under K management (Fig. 3b). In the first season, cotton boll number increased by K fertilization only when there was no ruzigrass. In the second season cotton boll number was higher than in the first year, the response to K was generalized, and even when K was not applied, more bolls were found after ruzigrass. Conversely, gin turnout was not affected by seasons, but was lower in the absence of K (Fig. 3c). In the first season the effect of K management on the low cotton yields was not consistent, but they were lower in plots without K fertilization (Fig. 3d). However, in the 2017/18 season, yields were higher under K fertilization irrespective of the presence of the grass and of K fertilization management. In the absence of K, cotton yield was 16% higher when ruzigrass was grown before cotton.

There was no significant interaction of years with treatments for plant height, number of nodes and percentage of short fibers. In the second season, cotton plants were taller, with more nodes and higher percentage of short fibers than in 2016/17 (Table 1); however, fiber strength and length were lower. Plant height responded to K application, and was highest when 116 kg ha^−1^ of K was applied with no ruzigrass, while the number of nodes was lower without K, irrespective of the grass. There was no effect of K fertilization or presence of the grass on fiber strength or length, but the percentage of short fibers was lower when there was more K available for cotton (Table 1).

**Table 1:** Yield components and fiber strength (STR), length (LEN) and short fiber content (SFC) of cotton cultivars as affected by year and potassium management.

	Plant height (cm)	Node (#)	STR (g tex^−1^)	LEN (mm)	SFC (%)
**Year**					
2016/17	81.7 b	12.1 b	31.3 a	29,6 a	7.0 b
2017/18	96.9 a	18.3 a	29.9 b	28,9 b	9.9 a
**Treatment**					
0 K	82.0 c	14.1 b	30.0	29.1	8.9 a
116 K	100.3 a	15.6 a	30.6	29.1	8.5 ab
0 K + RZ	84.2 c	14.6 b	30.3	29.1	8.6 ab
116 K on RZ	90.8 b	15.6 a	30.8	29.2	8.3 b
58 K on RZ + 58 K on C	91.7 b	15.7 a	31.1	29.4	8.2 b
116 K on C + RZ	87.1 bc	15.5 a	30.6	29.2	8.2 b
Year (Y)	0.001	0.001	0.001	0.001	0.001
Treatment (T)	0.001	0.001	0.06	0.57	0.11
YxT	0.22	0.03	0.16	0.73	0.79
CV(%)	10.67	8.64	3.66	2.18	10.78

*MIC* micronaire, *STR* strength, *LEN* length, *SFC* short fiber content.

### K fertilizer apparent use efficiency

**Figure 4:**
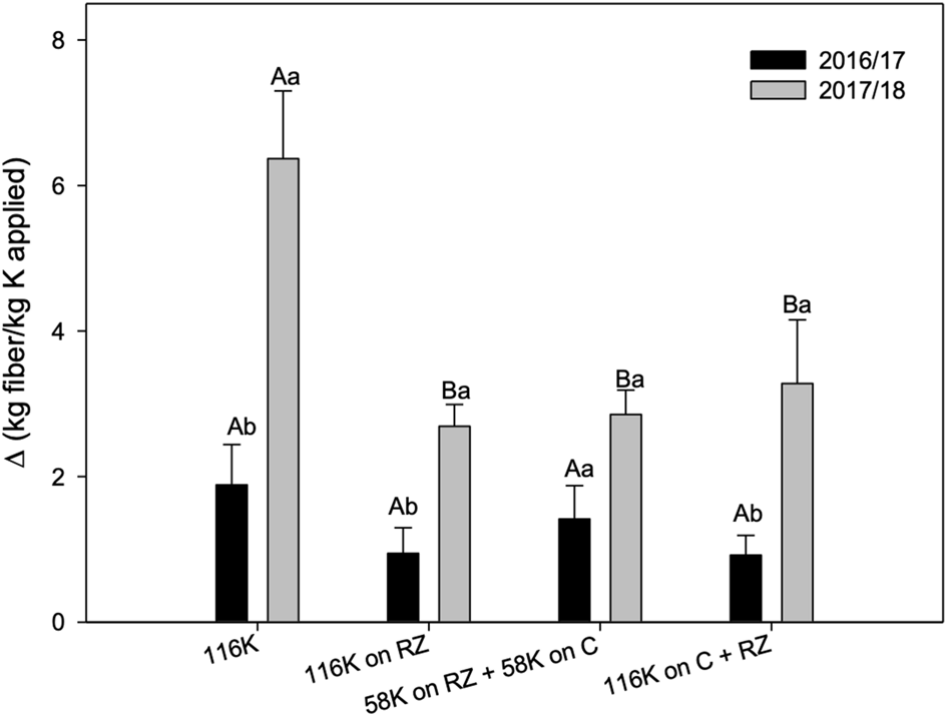
Potassium fertilizer apparent use efficiency (from fertilizer) represented by fiber production per kg of K applied. This index was obtained considering the difference of yield from treatments with no K (0 K for 116 K and 0 K + RZ for the others) divided by 116. A > B for K management within each growing season and a > b for growing season within K management.

The apparent K fertilizer use efficiency was lower when ruzigrass was grown before cotton in the season when the response to K was significant (2017/18), irrespective of the mode of application (Fig. 4).

### Fiber quality

**Figure 5:**
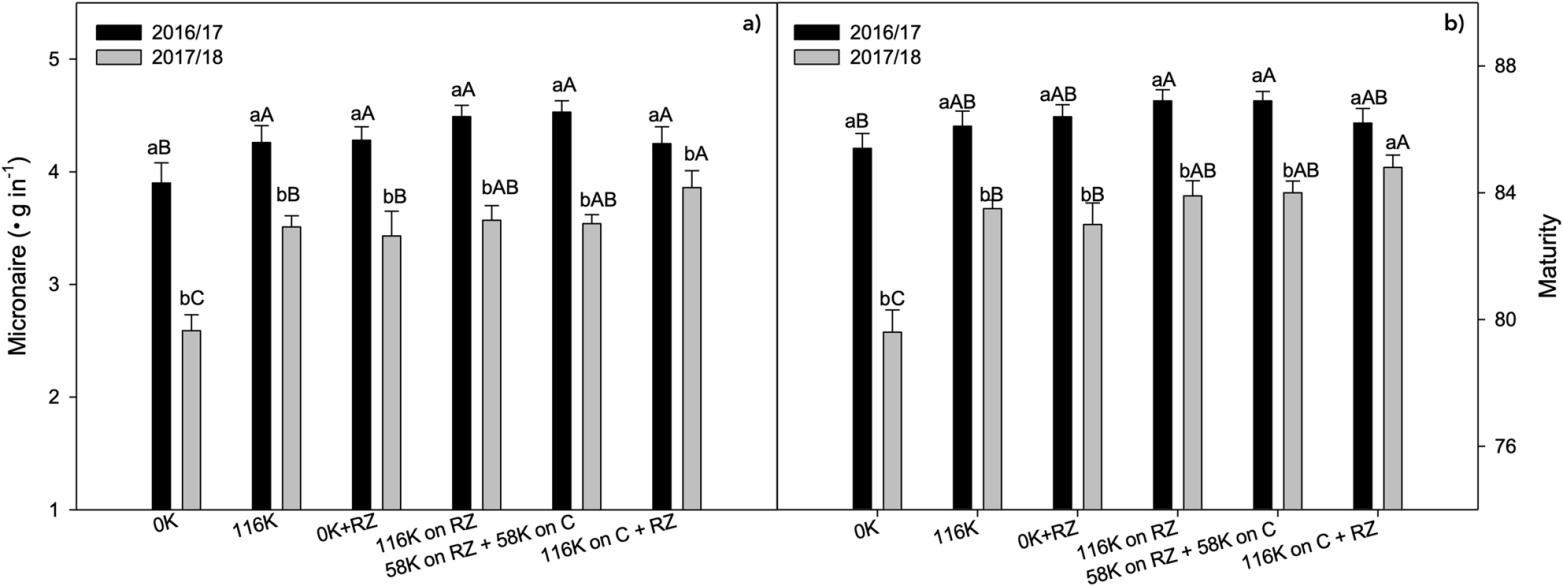
Micronaire (**a**) and fiber maturity (**b**) under potassium fertilization management. A > B for K management within each growing season and a > b for growing season within K management.

Interactions of the year with K management were significant for fiber micronaire and maturity (Fig. 5). On average, micronaire was lower in the second season. In both seasons, micronaire was lowest when no K was applied to plots without ruzigrass. It is noteworthy that just growing ruzigrass before cotton was enough to increase the fiber micronaire as much as applying 116 kg ha^−1^ of K without the grass. Fiber maturity responded to K in both years, and was higher in the first season in most treatments (Fig. 5b). Generally, it was higher when the K fertilizer was applied to the grass or split in the grass and sidedressed in cotton, with small and inconsistent differences between years for the other treatments.

### Pearson correlation coefficients

Most of the vegetative and yield components were positively related to each other (Table 2), but the correlation coefficients, despite being statistically significant (*P* < 0.05), were generally low, below 0.60, except for the relationships of node and boll number with fiber yield (Table 2). Conversely, fiber yield was generally negatively correlated (*P* < 0.05) with most of fiber quality parameters, but again the correlation coefficients, despite being statistically significant were low.

**Table 2:** Pearson correlation coefficient for plant height (H), node number (NN), boll weight (BW), boll number (BN), yield (Y), micronaire (MIC), fiber strength (STR), length (LEN), short fiber content (SFC) and fiber maturity (MAT).

	H	NN	BW	BN	Y	MIC	STR	LEN	SFC
NN	0.53*	–	–	–	–	–	–	–	–
BW	0.35*	0.30*	–	–	–	–	–	–	–
BN	0.53*	0.73*	0.07	–	–	–	–	–	–
Y	0.57*	0.76*	0.46*	0.90*	–	–	–	–	–
MIC	− 0.06	− 0.55*	0.24*	− 0.46*	− 0.28*	–	–	–	–
STR	− 0.27*	− 0.38*	− 0.09	− 0.23*	− 0.20*	0.39*	–	–	–
LEN	− 0.23*	− 0.35*	− 0.05	− 0.31*	− 0.25*	0.42*	0.64*	–	–
SFC	0.48*	0.67*	0.31*	0.45*	0.47*	− 0.54*	− 0.60*	− 0.74*	–
MAT	− 0.10	− 0.54*	0.24*	− 0.43*	− 0.24*	0.97*	0.55*	0.51*	− 0.60*

*P < 0.05.

## Discussion

Fiber yield was impaired in the 2016/2017 crop season (Fig. 3a, c; Sup. 2) by water deficit from the first square to the first flower, which probably increased square shedding and modifying boll distribution within the plant^18^. At boll maturation, the temperatures were low, impairing fruit development. Under these conditions, K application to cotton without ruzigrass resulted in higher fruit load and a higher yield than in the other K managements (Fig. 3b). Interestingly, in this season, soil K was higher after cotton harvest (Fig. 1). This could be the result of the retention of K on ruzigrass and cotton residues. The K applied and taken up by the grass depends on rainfall to be washed back to the soil^10^, and so it is retained longer in the straw in a dry spell, delaying K cycling and further impairing K uptake by cotton. With time, K was washed, which explains why soil K was lower than when there was no ruzigrass, but still higher than that observed in the second season. Previous research has shown that the crop and rainfall impact K release from plant residues to the soil, eventually affecting K recycling and also the temporal dynamic of K in the system^32^. In cotton, most of the K is stored on stalks before harvest (80% of total)^33^ and will be returned to the soil after shredding, which depends also on rainfall. With low rainfall, less K would have been leached from the tissue by the time the soil was sample, and this would help to explain the lower soil K in the second year, since plants were shorter, with fewer nodes in the first year, accumulating less K (Table 1).

The K concentration in the leaves was low (sufficiency range of 15–25 g kg^−1^) in the first crop season (Fig. 2). A dry spell was observed when cotton grow rate was supposed to experience a sharp increase, and so K uptake^19^. Considering that soil K transport to the root surface occurs by diffusion and mass flow^2,5^, it depends on soil water. It is hypothesized that the lower leaf K concentration was caused by the poor rainfall during flowering (for both early—45–60 DAE—and late stages—60–75 DAE) in the 2016/17 season. The effect of the lower water availability hindered both potassium solubilization and uptake, what help to explain the low K concentration on plant tissue and also the higher soil K in the first season**.** Hence, it can be inferred that cotton K uptake was impaired (Sup. 2), resulting in low K concentration in the leaves and eventually in low yield, which was aggravated by the low temperatures at the end of boll filling.

Splitting K fertilizer application has been recommended to avoid damage to the seedlings^20^, limit K fixation in minerals in clay soils^21^, and loss by leaching in sandy soils^8^. Yang et al.^22^ showed higher yields when K fertilization (90 kg ha^−1^) was split (50% at pre-planting and 50% at peak flowering) compared with full application in pre-planting in a high K soil. However, splitting K application showed no improvement in cotton fiber yield (Fig. 3d) or quality (Table 1) in the present experiment, where cotton was grown after ruzigrass. This can be explained, since there was no K close to the seed at planting time, and so, there was no damage to the seeds and seedlings. The nutrient that was taken up by ruzigrass was cycled back to the soil in time to meet cotton demand^10^ , without harming fiber yield and quality, since the smaller number of bolls observed when K application was split was compensated by a higher boll weight (Fig. 3).

**Figure 6:**
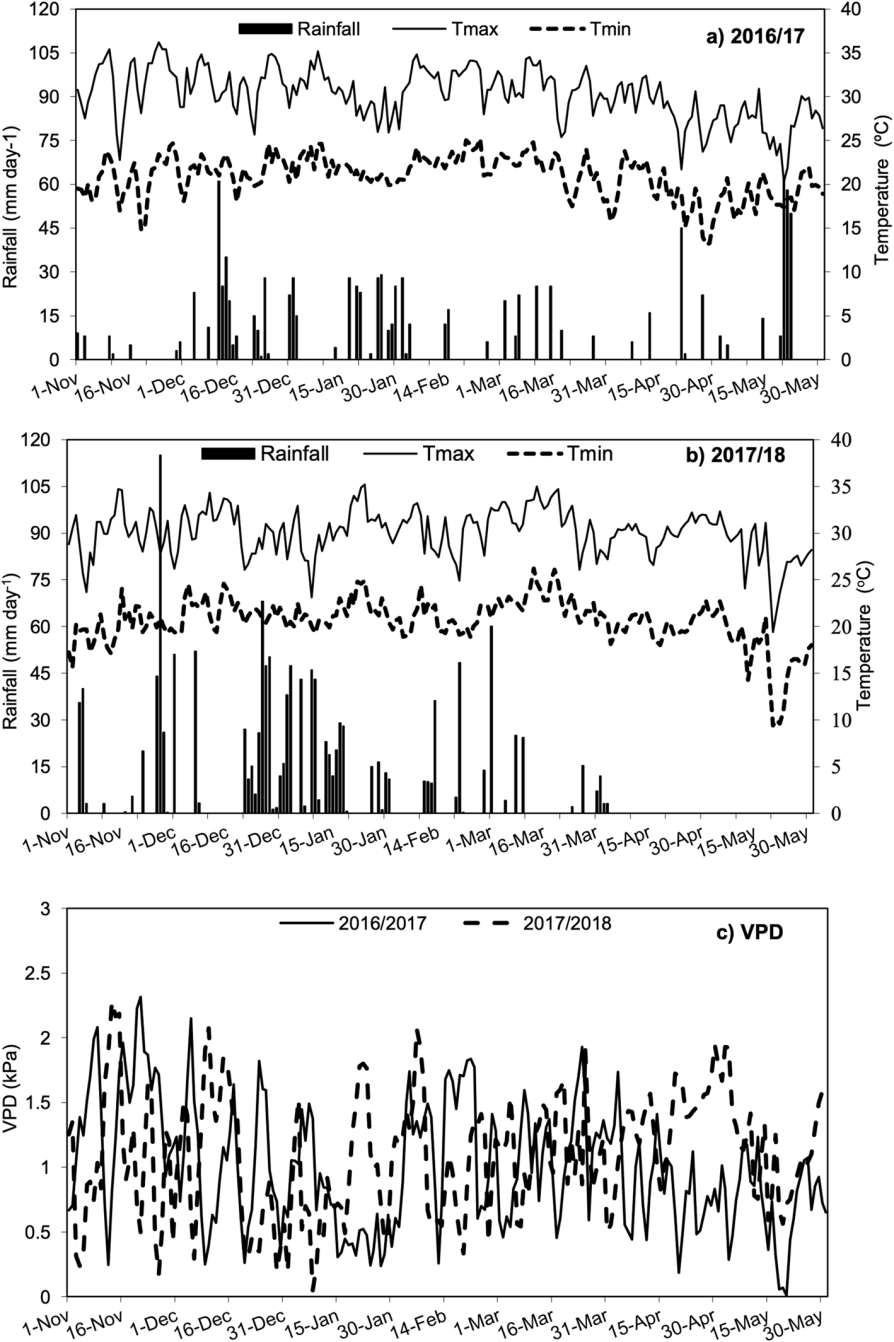
Rainfall, maximum and minimum temperatures, and vapor pressure deficit during the experiment.

The lower boll number in the 2016/2017 season was the result of the poor plant growth (height and number of nodes—Table 3) due to a water deficit from the first square up to the first flower, and low temperature at boll filling (Fig. 6), which also decreased cotton boll weight. However, due to the lower number of fruits (drains), the fiber quality of the remaining fruits was preserved^23^. Boll number was highly correlated with yield and node number, since fruiting branches sprout from the mainstem nodes. It is noteworthy that cotton yield response to K depended more on boll number than on boll weight (Table 2). This is important because when K is available to cotton late in the season, the effect on boll number is limited, since rainfall and temperatures are lower. This may have happened in the first season of this experiment due the delayed K cycling back to the soil.

**Table 3:** Selected chemical properties and particle size distribution of the soil.

Depth (cm)	pH (CaCl_2_)	SOM (g dm^−3^)	P (Resin) (mg dm^−3^)	S	Al^3+^	H + Al (mmol_c_ dm^−3^)	K^+^ (mmol_c_ dm^−3^)	Ca^2+^	Mg^2+^		CEC
0–20	4.7	13.6	2.8	4.0	0.6	17.6	0.8	7.3	5.1		30.7
20–40	4.8	11.6	2.0	3.3	2.3	18.6	0.8	6.5	4.4		30.3

	B (mg dm^−3^)	Cu (mg dm^−3^)	Fe (mg dm^−3^)	Mn (mg dm^−3^)	Zn (mg dm^−3^)	m (%)	BS (%)	Sand (g kg^−1^)	Silt (g kg^−1^)	Clay (g kg^−1^)	
0–20	0.34	2.0	26.0	0.9	0.4	4.3	42.6	848	36	116	
20–40	0.41	1.4	31.5	1.0	0.3	16.4	38.6	841	23	137

When all the fertilizer (116 kg ha^−1^) was applied to the grass, the lower soil exchangeable K content was consistent in both seasons. A similar result has been reported before in peanut fields but not for cotton under sod-based rotations with *Paspalum notatum*^24^. In the second season, cotton plants were taller, with a higher number of bolls, higher boll weight, higher yield, and higher K concentration in the leaves compared with the first season. Therefore, it can be inferred that plant dry weight and K uptake were also higher. This higher K demand explains why soil K decreased in the second season. Moreover, it can be inferred also that the K taken up by the grass was not returned in full to the soil because when K was applied to ruzigrass the soil K content was lower.

Apparently, the K fertilizer use efficiency was decreased by ruzigrass (Fig. 4), but it is important to consider that this grass has a high ability to take up soil K, and it is considered non-exchangeable^5^, and can accumulate as much as 400 kg ha^−1^ of K in stems and leaves^25^. As K is not metabolized and binds weakly with organic molecules^9^ it is available to be washed out of the straw after cover crops are terminated, which depends on the rainfall^10^. With the first rains, K is washed out of the straw, reaching the soil, it is available for the next crop. The use of grasses can be a strategy to improve potassium acquisition efficiency in modern agriculture, especially in low K soils, since the exudation of organic compounds releases non-exchangeable soil K, increasing the availability to other crops; grasses also have extensive root systems and high root-to-shoot ratios, with increased surface area for K uptake^26^. Therefore, when grown after ruzigrass, cotton plants had more K available than just that of the fertilizer. This inference is supported by the higher yield observed with ruzigrass without K fertilization (Fig. 3) as well as by the higher leaf K content, particularly in the second season (Fig. 2). Hence, the grass decreased cotton dependence on the fertilizer in the second season (Fig. 4), which resulted in less yield per unit of fertilizer K. It is tempting to speculate that K rates could be lowered after ruzigrass, but this could, with time, result in soil K depletion.

Low short fiber content (SFC) is a desired trait and it was reduced when cotton was K fertilized and grown after ruzigrass (Table 1). Though fiber length is most affected by genotype trait, SFC is dependent on genotype but also on growing conditions^27^. The lack of K tends to decrease fiber length^28^ as K is involved in osmoregulation and solute (malate and sugar) accumulation into fibers^22^, so the increased SFC was attributed to K shortage (Tables 3, 1).

Micronaire (MIC) was higher in the first season (Fig. 5) as a result of the lower fruiting sites per plant (Table 2; Fig. 3) plus the water deficit late in the season in 2017/2018 (Fig. 6), since an adequate water supply during the season allows for the maturation of more bolls at the upper canopy and outer fruiting positions^27^ (second and third positions). Also, the rise in MIC and fiber maturity on K fertilized with ruzigrass treatments in 2017/2018 may be associated with the ability of ruzigrass to enhance cotton potassium nutrition^13^. To our knowledge this is the first report of the additive effect of K and ruzigrass on cotton fiber micronaire and maturity.

## Conclusion

The early application of K fertilizer to ruzigrass grown before cotton improves the plant response to the nutrient because K is timely recycled, assuring a good K nutrition, yield and fiber quality, with no need to split K fertilization, what would be recommended in sandy soils without the ruzigrass in the system. As K loss is decrease, in the long-term it can allow for a reduction in K rates, lowering production costs and optimizing farm operation.

## Material and methods

A field experiment was conducted in the 2016/17 and 2017/18 cropping seasons at Presidente Bernardes, São Paulo State, Brazil, 22° 07′ 32″ S and 51° 23′ 20″ W, 475 m asl. The soil is a typic Rhodustult, sandy loam (USDA, 2010). The climate is Aw according to Köppen’s classification, tropical with dry winters and wet, hot summers. Soil samples were taken at the depths of 0 cm to 20 cm and at 20 cm to 40 cm on May 05, 2016, for chemical analysis^29^. Selected chemical and particle size distribution of the soil are in Table 3. Lime (45% of CaO and 4.9% of MgO) was broadcast on the soil surface at 1400 kg ha^−1^ in September 2016 and 2000 kg ha^−1^ in October 2017. Rainfall, maximum and minimum temperatures, and vapor pressure deficit during the experiment are shown in Fig. 6.

The treatments were an early (FM 913GLT) and a late (FM 983GLT) cotton cultivar and six K fertilization schemes (Table 4). Treatments were repeated in the same plots in both seasons (2016/2017 and 2017/2018). The experimental design was a 2 × 6 factorial in complete randomized blocks with five replicates. Each plot had four 7.0-m cotton rows spaced 0.8 m from each other. The two outer rows and 1.0 at the end of each row were considered borders and not included in evaluations.

**Table 4:** Scheme of cotton K fertilization.

Tratamentos	Ruzigrass (RZ)	K rate	Time of K fertilization
0 K	No	0	–
116 K	No	116 kg ha^−1^	58 kg ha^−1^ applied 30 DAE and 58 kg ha^−1^ at 45 DAE
0 K + RZ	Yes	0	–
116 K on RZ	Yes	116 kg ha^−1^	Applied on RZ vegetative stage (90 DAE)
58 K on RZ + 58 K on C	Yes	116 kg ha^−1^	58 kg ha^−1^ applied on RZ vegetative stage and 58 kg ha^−1^ on cotton 30 DAE
116 K on C + RZ	Yes	116 kg ha^−1^	58 kg ha^−1^ 30 DAE and 58 kg ha^−1^ 45 DAE

*DAE* days after emergence.

Ruzigrass was planted on May 05, 2016, and June 06, 2017 using 14 kg ha^−1^ of pure viable seeds. Potassium fertilizer was applied to the grass in the corresponding treatments early September each year, using potassium chloride. Ruzigrass was desiccated on November 1, 2016 and November 6, 2017, using glyphosate (1.92 g ha^−1^ a.i.), and cotton was planted on December 9, 2016, and November 23, 2017. Phosphorus was applied at 56 kg ha^−1^ and N at 30 kg ha^−1^ as mono ammonium phosphate at cotton planting. The rate of 140 kg ha^−1^ of N was sidedressed and split 35 and 45 days after plant emergence (DAE), using ammonium sulphate and urea, respectively. Boric acid was sprayed at 2.0 kg ha^−1^ split into 4 weekly applications from the first flower. Weed, pests, and diseases were controlled according to standard farm practices in São Paulo State. Seven days before harvest plants were defoliated with Tidiazurom (60 g ha^−1^ a.i.) + Diuron (30 g ha^−1^ a.i). Cotton was handpicked 141 and 132 DAE in 2016/17 and 2017/18, respectively.

At full flowering, the fourth/fifth leaf from the top was sampled from 10 plants per plot for foliar diagnosis^30^. The tissue was dried to constant weight in a forced air oven at 65 ºC, ground and K was determined using atomic absorption.

At harvest, plant height and the number of nodes per plant were determined in 5 randomly assigned plants per plot. Cotton yield, boll number and weight were determined by counting and weighing all bolls from the central 2.0 m of the inner cotton rows. A subsample was taken to be ginned, and the gin turnout, fiber strength, length, maturity, micronaire and short fiber index were determined using a High Volume Instrument^31^. After harvest, soil was sampled at 0–20 cm depth for exchangeable K analysis as in Raij et al.^29^. Potassium use efficiency (KUE) was calculated using Eq. (1) for treatments without ruzigrass and Eq. (2) for treatments with ruzigrass:

1 $$KUE=\frac{{Y}_{116K}-{Y}_{0K}}{116}$$

2 $$KUE=\frac{{Y}_{116K+RZ}-{Y}_{0K+RZ}}{116}$$

where KUE is the potassium use efficiency; Y_116K_ is the yield of the treatment fertilized; Y_0K_ is the yield of the treatment unfertilized; Y_116K+RZ_ represents the yield of all fertilized treatments with ruzigrass (RZ) (116 K on RZ; 58 K on RZ + 58 K on C and 116 K on C + RZ); Y_0+RZ_ is the yield of unfertilized treatment with ruzigrass; C is cotton and RZ is ruzigrass. Results were expressed as kg of fiber produced per kg of K applied.

After testing for homogeneity and normality, data were submitted to ANOVA. The experiment was arranged in a complete randomized block design with three fixed factors (K management, cotton cultivar, and years) and five replicates. The data were analyzed with three-way analysis of variance. Since no significant difference was observed for cotton cultivars, results were averaged across cultivars (Sup. 1) and Tukey’s test (*P* < 0.05) was used to determine significant differences. Pearson correlation coefficients (Minitab, State College, Pennsylvania) were determined between cotton growth, yield components and fiber quality parameters.

## Supplementary information

Supplementary information.
