# Supplementary material for: Potassium application to the cover crop prior to cotton planting as a fertilization strategy in sandy soils
Source: Sci Rep. 2020 Nov 23;10:20404. doi: 10.1038/s41598-020-77354-x (PMC7684316; doi:10.1038/s41598-020-77354-x)
Supplement: Supplementary file 1 — Supplementary information. [file 41598_2020_77354_MOESM1_ESM.pdf]

Supplementary information

Potassium application to the cover crop prior to cotton planting as a fertilization strategy in sandy soils

Fábio Rafael Echer[a*]; Vinicius José Souza Peres[a]; Ciro Antonio Rosolem[b]

[a]Department of Agronomy, Universidade do Oeste Paulista. Raposo Tavares HWY, Km 572, 19067-175, Presidente Prudente, São Paulo, Brazil.[b] São Paulo State University, Botucatu, São Paulo, Brazil. *Corresponding author: fabioecher@unoeste.br

Supplementary information 1. The P-values for sources of variation ant their interactions for yield, yield components, fiber quality parameters, potassium on leaves and soil and potassium use efficiency.

Source of variation	BW	BN	GT	Y	Heigth	NN	STR	LEN	SFC	MIC	MAT	Kleaf	K soil	KUE
Year (Y)	0.001	0.001	0.001	0.001	0.001	0.001	0.001	0.001	0.001	0.001	0.001	0.001	0.001	0.001
K manag.(K)	0.001	0.001	0.001	0.001	0.001	0.001	0.06	0.57	0.11	0.001	0.001	0.001	0.46	0.001
Cultivar (C)	0.001	0.01	0.001	0.85	0.001	0.71	0.007	0.52	0.001	0.001	0.001	0.54	0.001	0.09
YxK	0.009	0.08	0.002	0.001	0.22	0.03	0.16	0.73	0.79	0.01	0.001	0.05	0.04	0.04
YxC	0.78	0.54	0.17	0.92	0.47	0.34	0.001	0.001	0.43	0.01	0.001	0.35	0.10	0.23
KxC	0.14	0.17	0.52	0.16	0.31	0.72	0.06	0.23	0.24	0.60	0.27	0.23	0.28	0.16

BW: boll weight; BN: boll number; GT: gin turnout; Y: yield; NN: node number; STR: fiber strenth; LEN: fiber length; SFC: shrot fiver contente; MIC: micronaire; MAT: maturity; KUE: K use efficiency.

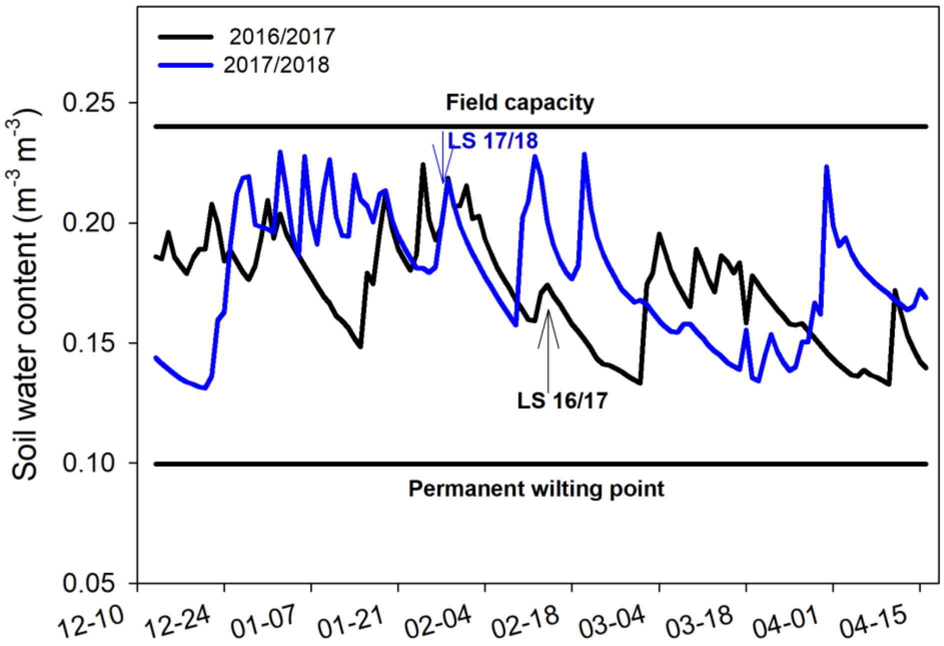

Supplementary information 2. Soil water content (0-50 cm depth) during 2016/17 and 2017/18 seasons. Leaf sampling (LS) is indicated by black (2016/17) and blue arrows (2017/18).
